# A Rare Case of Recurrent *Klebsiella pneumoniae* Liver Abscess

**DOI:** 10.1155/2021/8896379

**Published:** 2021-04-09

**Authors:** Magdelene Amoateng, Pius Osei-Bagyina, Reba Varughese, Achsah Mathew, Ishan Malhotra

**Affiliations:** ^1^Department of Internal Medicine, Quinnipiac University Frank H. Netter MD School of Medicine, St. Vincent's Medical Center, Bridgeport, CT, USA; ^2^Pushpagiri Institute of Medical Sciences & Research Center, Tiruvalla, Kerala, India; ^3^St. Vincent's Medical Center, Bridgeport, CT, USA

## Abstract

*Klebsiella pneumoniae* liver abscess (KPLA) is an emerging syndrome with the initial cases described in Taiwan in the 1980s. There is high mortality with this condition, and immediate aggressive treatment is necessary. Diabetes mellitus (D.M.) is the single most important risk factor for developing KPLA. Here, we describe a rare case of recurrent cryptogenic *Klebsiella pneumoniae* pyogenic liver abscess (KPLA) in a young man with poorly controlled type 1 D.M.

## 1. Introduction

A pyogenic liver abscess (PLA) was once thought to be a polymicrobial infection with *E. coli* as the primary causative organism in those with underlying hepatobiliary tract diseases and gastrointestinal pathologies [1]. Recently*, Klebsiella pneumonia* has been found to be an emerging cause of invasive syndrome of monomicrobial cryptogenic pyogenic liver abscess (PLA) in the absence of underlying hepatobiliary pathology [2]. Diabetes mellitus is recognized as the single most important risk factor for developing *Klebsiella pneumoniae* liver abscess (KPLA), with poor glycemic control contributing to enhanced pathogenicity of *Klebsiella pneumoniae* [3]. The infection is associated with high mortality and life-threatening metastatic complications. It is increasingly recognized in the United States and is more commonly found in Asian and Hispanic populations in the USA [4]. However, recurrent KPLA is rarely described in the literature. Here, we describe a rare case of recurrent cryptogenic *Klebsiella pneumoniae* liver abscess in a young man with poorly controlled type 1 D.M.

## 2. Case Report

**Figure 1:**
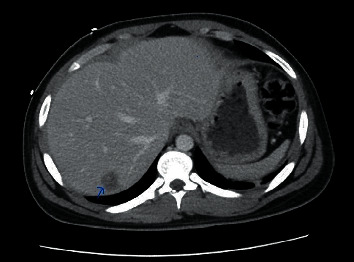
C.T. abdomen revealing abscess in the 7th segment of the liver.

**Figure 2:**
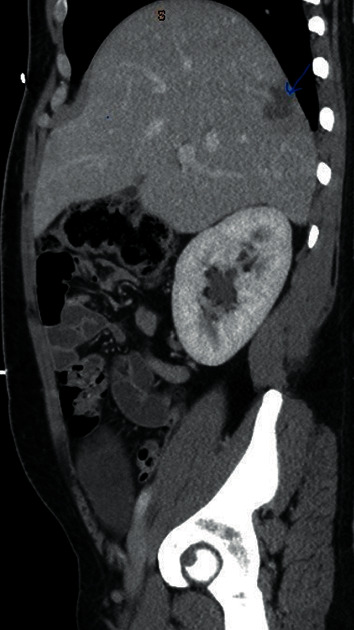
Sagittal view of the abdomen revealing the liver abscess.

**Figure 3:**
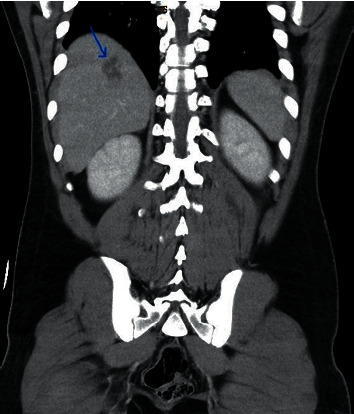
C.T. abdomen coronal view revealing the liver abscess.

**Figure 4:**
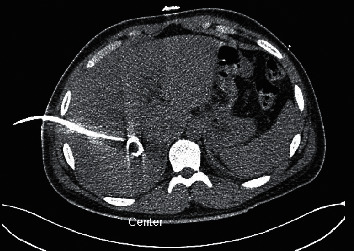
C.T. abdomen revealing the drain that was placed for the liver abscess.

**Figure 5:**
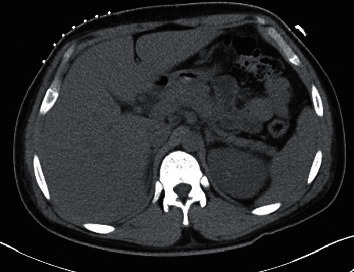
C.T. abdomen after drainage.

This is a 20-year-old man from Guatemala with a history of poorly controlled type 1 D.M. and 2 hepatic abscesses: the first drained in Guatemala 6 years prior and the second drained 4 months in the US, prior to the current presentation. It is unclear what was isolated in the first event, but he was treated for *Klebsiella pneumoniae* liver abscess with antibiotics after the second drainage. There were no previous images for comparison. He presented with a 2-day history of altered mental status, high fever, abdominal pain, and occipital headache. He was found to be septic with diabetic ketoacidosis and acute hepatitis. C.T. of the abdomen showed a heterogeneous rounded low-attenuation lesion with irregular borders in the posterior segment of the liver's right lobe (segment 6) (Figures 1–3) and three small nodular opacities in the right lung, which appeared infectious. Laboratory data were significant for leukocytosis with severe lactic acidosis. Blood culture was positive for *Klebsiella pneumoniae*. He underwent guided drainage of the abscess by interventional radiology (Figures 4 and 5), and the culture of the fluid isolated the same organism. He was treated with antibiotics, intravenous fluids, and insulin therapy with improvement in his symptoms. The patient was discharged on long-term antibiotics (7 weeks) and insulin therapy, and he was counseled on medication adherence to achieve strict glycemic control.

## 3. Discussion

*Klebsiella pneumoniae* PLA is an emerging invasive syndrome with the first cases described in Taiwan in the 1980s [5, 6]. *Klebsiella pneumoniae* is a Gram-negative bacillus. The pathogenesis of KPLA is not completely understood. The underlying hepatobiliary and gastrointestinal abnormalities such as gallstones, strictures, surgery, trauma, colorectal cancer, and infections contribute to the pathogenesis of PLA [1]. Interestingly, KPLA is usually cryptogenic, without any underlying pathologies. It is believed to result from the translocation of the organisms from the gut to the liver [7]. The most virulent strains identified are the K1 and K2 serotypes with *rmp*A and *mag*A genes, with hypermucoid and hypercapsular phenotypes. Over the past two decades, it is globally emerging as the primary causative agent for pyogenic liver abscess [4]. The reported incidence in the United States is 41% [4], with the highest predominance in Asian and Hispanic populations, even though a higher incidence of 80–90% is reported in Taiwan [6]. Currently, there is no evidence for the cause for this predominance among this population, and a genetic predisposition is not described. Recurrent *K. pneumoniae* PLA is rarely described. The average duration between the first episode and recurrent episode was 7.6 years with a range of 2 to 20 years [3]. Our patient had a second episode 6 years after the first episode of KPLA and four months later with the third episode. Diabetes mellitus, impaired fasting blood glucose, and recent antibiotic use are the commonly recognized predisposing factors. Poor glycemic control results in impairment of neutrophilic phagocytosis of K1/K2 *K. pneumoniae*, making it an important risk factor [8]. Approximately 50–75% of the patients with KPLA were found to have D.M. as a comorbid condition [2].

The typical clinical manifestations include fever, chills, and right upper quadrant tenderness. Nonspecific symptoms such as anorexia, nausea, vomiting, diarrhea, jaundice, and right-sided pleural effusion have also been reported [4]. Some patients may also present with bacteremia, sepsis, disseminated intravascular coagulation, acute respiratory distress, and acute renal failure [7].

Leukocytosis, thrombocytopenia, anemia, high C-reactive protein, erythrocyte sedimentation rate, hypoalbuminemia, elevated alkaline phosphatase, alanine aminotransferase (ALT), and bilirubin are the common laboratory findings [4]. Diagnosis requires abdominal imaging by ultrasonography (USG) or computed tomography (C.T.). *K. pneumoniae* PLA may appear as solid, hypoechoic nodules on U.S. imaging and little pus with abundant necrotic material on aspiration [9]. Abdominal C.T. is preferred over USG due to increased sensitivity (100%). C.T. with contrast typically shows a thin-walled single, multiseptated, solid mass with a necrotic area [10]. The majority of the liver abscesses are found in the right lobe, primarily due to its size and dense hepatic tissue, the rich blood supply from the portal vein, and the network of bile canaliculi [7].

Metastatic complications include bacteremia, meningitis, endophthalmitis, septic pulmonary emboli, and necrotizing fasciitis, of which endophthalmitis is the most commonly reported metastatic complication that can be sight-threatening. The overall mortality is 5% [11], which increases to approximately 10–16% when presenting with metastatic infections [12].

Diabetes is responsible for the recurrence of the KPLA. In a study conducted by Ya-Sung et al., six cases of recurrent KPLA were studied. Most of the cases had diabetes and the K1 serotype of *Klebsiella* [3]. Isolates were uniformly susceptible to cefazolin, and distinct molecular fingerprints were found for the strains isolated from both primary and recurrent KLAs [3]. Poor glycemic control has been attributed to the phagocytic resistance against *K. pneumoniae* mediated by the capsular K-antigen, which has increased the lethality of this serotype [3].

Percutaneous drainage along with antibiotics is the mainstay of treatment. Third- and fourth-generation cephalosporins are the primary choice in the treatment of KPLA [4, 7].

Aminoglycosides and carbapenems are also used [7, 13]. The optimal duration of intravenous (IV) antibiotic therapy and subsequent oral therapy remains unclear. The study in Taiwan leads to a nearly standardized treatment with 3 weeks of IV antibiotics followed by 1 or 2 months of oral antibiotics [6]. However, studies in the US demonstrate extremely low mortality with a shorter duration of IV antibiotics (mean of 17.5 days) and oral antibiotics (mean of 13.6 days) [4]. Surgical drainage may be required in those patients with thick pus which cannot be aspirated, ongoing sepsis even after percutaneous drainage and antibiotic therapy, multiloculated abscess, multiple abscesses, or ruptured abscess [4, 7]. Strict glycemic control is essential to decrease metastatic complications.

## 4. Conclusion

The diagnosis of *Klebsiella pneumoniae* pyogenic liver abscess should be considered in patients with sepsis and bacteremia with comorbid D.M. In culture-positive patients with a poor response to antibiotic therapy, it is reasonable to search for occult liver abscesses as well as other metastatic complications. A high index of suspicion is required for prompt diagnosis and management to reduce the life-threatening complications associated with this condition.
